# Wake Up, Sleepers! The Time Has Come

**Published:** 1912-11-16

**Authors:** 


### The Hospital

The Workers' Newspaper of Administrative Medicine and Institutional Life, Administration, National Insurance and Health.

No. 1376, Vol. LIII. SATURDAY, NOVEMBER 16, 1912. PRICE ONE PENNY.

### WAKE UP, SLEEPERS! THE TIME HAS COME.

Last week and the week before we took pains to set clearly before our readers the actual position of matters with regard to the relations and difficulties which will arise for voluntary hospitals when the National Insurance Act begins to confer benefits upon the insured. The articles which have been published have been written from the point of view of accurate information and full knowledge. The managers of voluntary hospitals to-day have well within their reach a wise solution of all their difficulties, with the assured certainty that if they act with intelligent promptness the voluntary hospitals can be preserved and strengthened. The intelligent observer, if he were to judge by the spirit of inaction which seems abroad, with the absence of any sign of interest or movement in the quarters where these vital interests are at stake, might easily persuade himself that those directly responsible have taken to their beds and entered upon a long and enduring sleep. What is happening is, however, that individual hospitals are considering these questions in all their bearings, so that the time should not be far distant when, as we hope, the lethargy of the moment may be shed. The practical plan we have expounded of *pro rata* payments to replace the actual sum expended by each voluntary hospital upon insured persons, plus the remuneration of the medical staff of hospitals, and the institution of a new financial basis by which all capital sums required in future for buildings and extensions will be readily available to the fullest extent necessary whenever required, has been received not only with sympathy but with approbation in high quarters.

The alternative to the *pro rata* scheme, and it is the only alternative which has been voiced so far in this matter, is one which would destroy the voluntary hospitals and ensure their speedy absorption by the municipality or the State, or both. The proposal in question is that the exact sum of money by which the expenditure at a voluntary hospital for the past year had exceeded the income for that year should be ascertained, and that the State should hand to the hospital authorities a cheque for that excess, so that each year each hospital would commence again without any debt. The author of this proposal, apparently to make it attractive to the State, suggests that the State should appoint inspectors to make examination each year into the position, management and working of each participating hospital and its needs for the future. We need not spend time upon this latter proposal, for voluntary hospitals would not suffer it. As to the first, it must be patent to every thinking man of business that directly a voluntary hospital with a large deficit had that deficit made good by the State or the municipality, it would speedily cease to be a voluntary institution, if it did not even cease to exist. Who of all the subscribers and supporters of such an institution, after paying his quota on behalf of his employees under National Insurance, would sit down and draw a cheque in favour of the voluntary hospital in his district, knowing full well that the use of that cheque would be merely to diminish by its amount the total sum which the State or the municipality would otherwise supply? There are many other points which might be made, but the one just urged is fatal to this proposal.

The *pro rata* plan leaves the voluntary system untouched; it perpetuates its existence on a sound financial basis; it conserves the advantages and safeguards afforded to the patients compared with all other hospital systems; and must prove, from all points of view, to be economical, sound, and best for all classes. We are in a position to say that, providing the hospital managers will wake up and show alertness and judgment by preventing all avoidable delay, the road is open to them, and everything is ready to make the change from the old to the new plan of administration. It is further open to them under this system to secure all its financial advantages for the hospitals, all its safeguards for the patients, and an adequate recognition of the just claims for payment of the hospital medical staff of all grades. The tide of the voluntary hospitals is at the flood. Will the managers take it or lose it? Now is the time for action. Let all the friends of the voluntary hospitals wake up and be alive, whilst the managers go actively about their business. For the time has come when every hospital manager should realise, "Who seeks, and will not take when once 'tis offered, shall never find it more."

